# Ketosis regulates K+ ion channels, strengthening brain-wide signaling disrupted by age

**DOI:** 10.1162/imag_a_00163

**Published:** 2024-05-08

**Authors:** Helena van Nieuwenhuizen, Anthony G. Chesebro, Claire Polizu, Kieran Clarke, Helmut H. Strey, Corey Weistuch, Lilianne R. Mujica-Parodi

**Affiliations:** Department of Physics and Astronomy, Stony Brook University, Stony Brook, NY, United States; Athinoula A. Martinos Center for Biomedical Imaging, Massachusetts General Hospital and Harvard Medical School, Charlestown, MA, United States; Department of Biomedical Engineering, Stony Brook University, Stony Brook, NY, United States; Renaissance School of Medicine, Stony Brook University, Stony Brook, NY, United States; Department of Physiology, Oxford University, Oxford, United Kingdom; Department of Medical Physics, Memorial Sloan Kettering Cancer Center, New York, NY, United States; Laufer Center for Physical and Quantitative Biology, Stony Brook University, Stony Brook, NY, United States

**Keywords:** ketone ester, metabolism, synchrony, aging, multiscale modeling

## Abstract

Aging is associated with impaired signaling between brain regions when measured using resting-state functional magnetic resonance imaging (fMRI). This age-related destabilization and desynchronization of brain networks reverses itself when the brain switches from metabolizing glucose to ketones. Here, we probe the mechanistic basis for these effects. First, we confirmed their robustness across measurement modalities using two datasets acquired from resting-state EEG (*Lifespan:*standard diet, 20–80 years, N = 201;*Metabolic:*individually weight-dosed and calorically-matched glucose and ketone ester challenge,$\mu_{age}$= 26.9±11.2 years, N = 36). Then, using a multiscale conductance-based neural mass model, we identified the unique set of mechanistic parameters consistent with our clinical data. Together, our results implicate potassium (K^+^) gradient dysregulation as a mechanism for age-related neural desynchronization and its reversal with ketosis, the latter finding of which is consistent with direct measurement of ion channels. As such, the approach facilitates the connection between macroscopic brain activity and cellular-level mechanisms.

## 1 Introduction

Endogenous ketone bodies, includingD-β-hydroxybuty-rate (D-βHB), are produced by the liver and can be utilized by cells as fuel when glucose is not readily available (Krebs, 1960). Accumulating evidence suggests that ketone metabolism may confer important neurological benefits (Barañano & Hartman, 2008;Henderson et al., 2009;Maalouf et al., 2007;Vanltallie et al., 2005).D-βHB has been found to increase ATP production and cardiac efficiency from 10% to 24% when added to a perfusion of glucose (Sato et al., 1995). Moreover, ketone uptake bypasses the insulin-dependent glucose transporter GLUT4, and thus can be metabolized as fuel even after neurons become insulin resistant (Sapolsky, 1986).

Insulin-independent metabolic pathways may be critical in the context of brain aging, as Type 2 diabetes mellitus and its associated decrease in GLUT4-dependent neuronal glucose utilization are linked to age-related brain hypometabolism (Baker et al., 2011;Soares et al., 2019) and cognitive decline (Antal et al., 2022;Beeri et al., 2004). Even in the face of impaired glucose metabolism, aging brains can still metabolize ketone bodies (Cunnane et al., 2016). Furthermore, ketones also influence cerebral metabolism by modulating glucose uptake in astrocytes (Valdebenito et al., 2016) and directly affecting neuronal excitability (Cauli et al., 2023;Karagiannis et al., 2021). Thus, supplementing ketone bodies as an alternative fuel source may have the potential to slow or arrest neurodegeneration (Zilberter & Zilberter, 2017).

Recent findings provide evidence supporting the ability of exogenousD-βHB to ameliorate mechanisms and biomarkers associated with brain aging. At the*spiking-neuron*scale, insulin and ketone bodies modulate neuronal excitability through the regulation of K^+^ion gradients (Ma et al., 2007;Sweeney & Klip, 1998). At the*circuit*scale, direct application ofD-βHB to the mouse hippocampal CA3-CA1 circuit reverses insulin resistance-induced deficits in neuronal excitability and axon conduction velocity, showing improvements that exceeded even baseline (control) values (Kula et al., 2024). Finally, at the*whole-brain*scale, aging has been linked to the destabilization (Mujica-Parodi et al., 2020) and desynchronization (Weistuch et al., 2021) of brain networks identified in resting-state functional magnetic resonance imaging (rsfMRI). Ketosis, induced by either diet or exogenousD-βHB administration, improved these measures of brain-wide coordination, even in young, healthy adults. Despite the theoretical relationships suggested between these independent results, the direct linking across scales—of mechanism with its emergent effects—has yet to be tested due to the technical constraints inherent in each experimental approach.

**Fig. 1.**
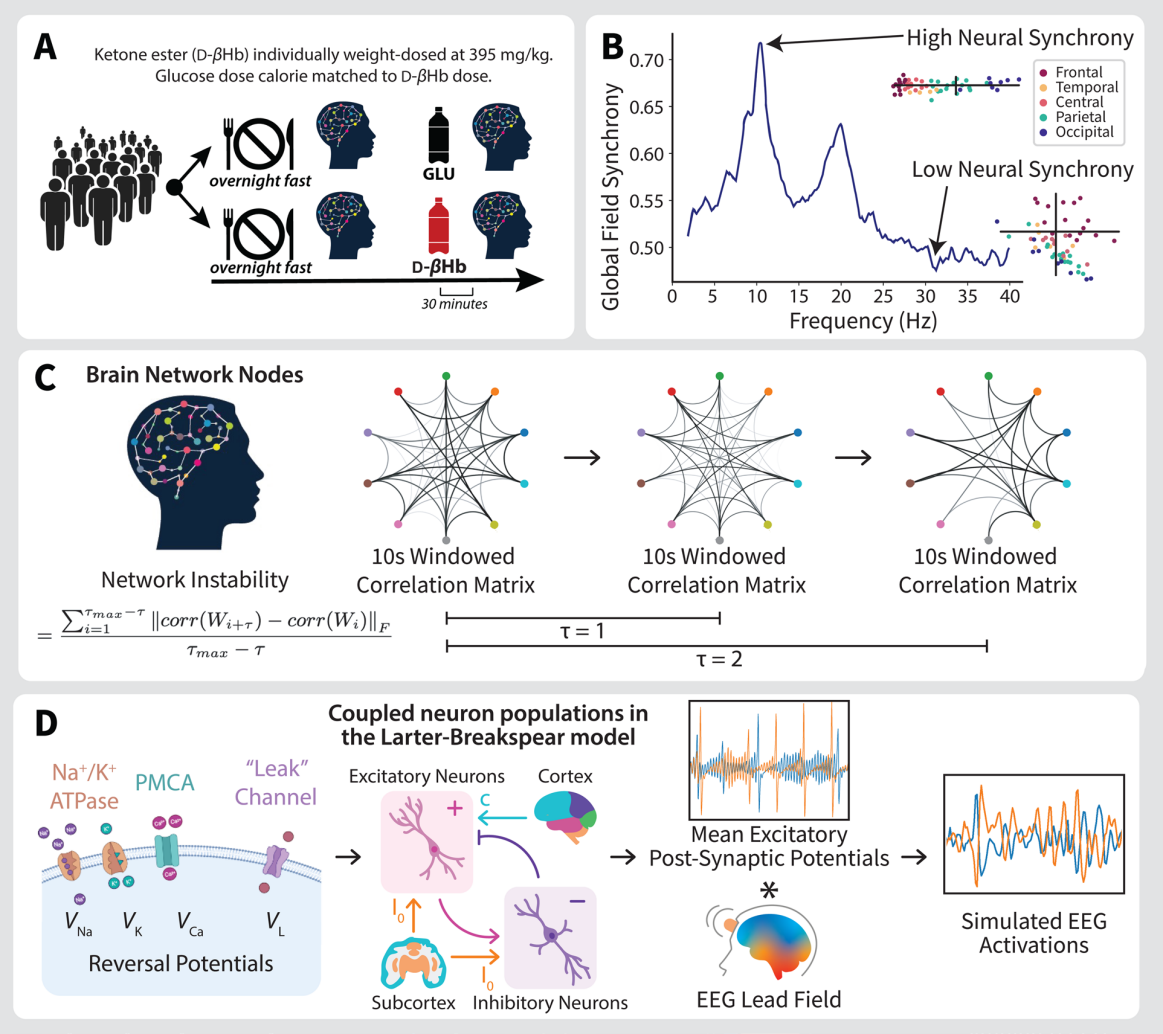
Schematic of experimental design and methods. (A) Design of within-subject, time-locked targeted metabolic resting-state EEG (rsEEG) experiment. To confirm the robustness of increased brain network instability and synchrony across measurement modalities, N = 36 young ($\mu_{age}$= 26.9±11.2 years), healthy participants underwent four rsEEG scans separated over 2 days. Following an overnight fast, participants were scanned at baseline and again 30 minutes after consuming a weight-dosed (395 mg/kg) ketone ester or calorie-matched glucose bolus. The rsEEG scans were then repeated using the opposite (ketotic or glycolytic) condition on the second day. (B) An example Global Field Synchronization (GFS) spectrum computed using human rsEEG. The real and imaginary components of Fourier-decomposed rsEEG time-series are plotted on the complex plane for each frequency value. The spread of these points is quantified using principal component analysis. The more the signals are in phase or anti-phase, the greater the difference in magnitude between the first and second principal components of the scatter plot cloud, and the greater the synchrony value (which can range from 0 to 1). The scatter points of individual electrodes are color-coded by location on the scalp for illustrative purposes. (C) Schematic characterization of brain network instability. To calculate brain network instability, non-overlapping sliding window correlations are calculated over the entire rsEEG time-series, with strong correlations defining networks. The instability of the networks is then defined as the degree to which these networks fluctuate over time (in units ofτ). (D) Schematic of the conductance-based neural mass model. Microscale parameters (listed inTable 1), along with intra-/inter- region coupling (c) and subcortical excitatory inputs ($I_{0}$) govern the dynamics of the model output: simulated excitatory post-synaptic potentials (EPSPs). The mean EPSPs are multiplied with an EEG lead field to generate simulated EEG time-series, which are used to determine the effects of model parameter variation on synchrony.

To address these challenges, we employ a novel experimental-computational approach for evaluating the contribution of individual biophysical components on brain-wide coordinated behaviors. To link fMRI-derived brain network instability and synchrony specifically to the postsynaptic potentials of pyramidal neurons, we established an EEG-derived biomarker for brain aging by analyzing a publicly available*Lifespan*resting-state EEG (rsEEG) dataset (ages 20-80 years,N=201). To test how this biomarker changes with metabolism, we conducted a new*Metabolic*rsEEG experiment ($\mu_{age}=\;26.9$±11.2years,N=36) (Table S1) (Fig. 1A). Administering individually weight-dosedD-βHB versus calorically-matched glucose orally, we measured the effects of fuel type on synchrony (Fig. 1B) and brain network instability (Fig. 1C). To identify the set of mechanistic parameters consistent with our data, we then used our EEG results as emergent constraints on the Larter-Breakspear model, a conductance-based multiscale neural mass model (Breakspear et al., 2003). By simulating this model across a full range of key mechanistic parameters and simultaneously testing$3^{8}$hypotheses in parallel (three types of trends, eight parameters), we were able to test whether measured differences in EEG-derived neural synchrony at the macroscale were consistent with effects expected following modulation of K^+^at the neuronal micro-scale (Fig. 1D). Equally important, however, by testing alternative plausible hypotheses in parallel, we were able to assess not only the sensitivity but also the specificity of our results.

## 2 Methods

### 2.1 Bolus study experimental design

To determine whether stabilization of brain networks as modulated by fuel source seen in rsfMRI (Mujica-Parodi et al., 2020;Weistuch et al., 2021) implicates neuronal synchrony, we conducted resting-state EEG scans on a cohort of healthy adults (N=36,$\mu_{age}=26.9\pm 11.2$years, 18 female) who were tested under both a ketotic (ketone burning) and glycolytic (glucose burning) condition. Inclusion/exclusion criteria were screened using a survey completed before enrollment in the study. Potential participants were excluded for any of the following reasons: chronic usage of alcohol (7 drinks/week for women, 14 drinks/week for men), recreational drug use, use of psychotropic medication within the past 30 days, use of medications that affect glucose and/or insulin utilization, a history of kidney disease, heart attack, stroke, myxedema, epilepsy, dementia, or other neurological disorders, difficulty when swallowing, current pregnancy or breastfeeding, or inability to provide informed consent. Scanning occurred at Stony Brook University’s Health Science Center. The study was approved by the Institutional Review Board of Stony Brook University (IRB2021-00018), and all participants provided informed consent.

All subjects were tested twice (1–14 days apart), both times following an overnight fast (subjects were instructed to eat no food for at least 8 hours before testing but were allowed unrestricted water). Following a baseline resting-state EEG scan, subjects drank either of two fuel sources. In the ketotic session, subjects drank a ketone sports drink, deltaG® Sports Supplement (TdeltaS Ltd, Thame, UK), dosed at 395 mg/kg. During the glycolytic session, the same subjects drank a bolus of glucose (Glucose Tolerance Test Beverages, Fisher Scientific, Inc.; Hampton NH) calorie-matched to the ketone bolus. The order of the bolus (whether a subject received a glucose bolus first or a ketone bolus first) was pseudo-randomized, with approximately half of all female subjects (N=9) and exactly half of all male subjects (N=8) drinking the glucose bolus during the first scanning day. The resting-state EEG scans were then repeated 30 minutes after the administration of the bolus, as prior experiments using magnetic resonance spectroscopy (MRS) showed peak glucose and ketone concentration in the brain approximately 30 minutes after consumption of the bolus (seeFig. S2inMujica-Parodi et al. (2020)). Blood glucose and ketone levels were measured three times throughout the experiment: at baseline, 10 minutes following the bolus, and 62 minutes following the bolus using a Precision Xtra Blood Glucose & Ketone Monitoring System (Abbott Laboratories). Our data analyses quantify network reorganization and neural phase synchrony changes in response to changing energy constraints (i.e., cognitive demand, fuel).

During the resting-state portion of the EEG scan, subjects underwent a total of 16 blocks, each lasting 60 seconds, with 8 EO blocks and 8 EC blocks. The blocks were interleaved. During the EO blocks, subjects fixated on a white orienting cross on a black background. Before the resting-state scan, subjects were instructed not to blink to minimize ocular artifacts and to keep motion to a minimum. The resting-state stimulus (a white cross in the center of a black background) was presented on a computer screen placed in front of the seated subject using PsychoPy v3.0 (Peirce, 2007). All data were collected in a shielded, dark, soundproofed Faraday cage using the ActiveTwo Biosemi™ electrode system from 65 (64 scalp, 1 ocular) electrodes arranged according to the international standard 10–20 system (Oostenveld & Praamstra, 2001) at a sampling frequency of 4096 Hz. The ocular (VOEG) electrode was placed below the left eye. Our experimental and pre-processing designs, especially those of the resting-state EEG scans, were modeled after the paradigm used by Leipzig’s LEMON rsEEG dataset group (Babayan et al., 2019) to be able to minimize confounding factors when directly comparing results between our experiment and this large-scale neuroimaging dataset.

### 2.2 Resting-state EEG pre-processing

#### 2.2.1. Metabolic dataset

The EEG pre-processing was performed using EEGLab (version 2020.0) (Delorme & Makeig, 2004). Full resting-state data were downsampled from 4096 Hz to 512 Hz and bandpass filtered between 0.1 and 40 Hz using a Hamming-windowed FIR filter. The data were then separated into the eyes open (EO) and eyes closed (EC) conditions. These two conditions were pre-processed separately from this point on due to the differences in ocular artifacts in each condition; however, the pre-processing steps performed were identical. Bad channels were removed, and noisy portions of data were identified and removed using EEGLab’s Artifact Subspace Reconstruction (ASR) algorithm. Independent component analysis (ICA) was performed on the data using the infomax algorithm in EEGLab (runica), and non-neural components of the time-series were identified and removed using ICLabel (Pion-Tonachini et al., 2019). The reference was then set to average. The data were separated into frequency bands and time-series extracted using MNE-Python (version 0.21.1) (Gramfort et al., 2013).

#### 2.2.2. Leipzig LEMON dataset

Lifespan rsEEG data from the Leipzig LEMON dataset were obtained in already pre-processed form. Raw data were downsampled from 2500 Hz to 250 Hz and bandpass filtered between 1 and 45 Hz using an 8th order Butterworth filter. The data were separated into EO and EC conditions for subsequent pre-processing. Outlier channels were rejected, and data intervals containing high peak-to-peak fluctuations or high-frequency noise were identified and removed by visual inspection. Data dimensionality was reduced using principal component analysis before the use of independent component analysis (ICA) to identify and remove components reflecting eye- or heartbeat-related artifacts. Further pre-processing details may be found in the dataset documentation (Babayan et al., 2019).

### 2.3 Global field synchronization

Global Field Synchronization (GFS) was first introduced byKönig et al. (2001)to estimate differences in functional connectivity of brain processes in EEG frequency bands between a population of neuroleptic-naive schizophrenic patients and healthy controls. In contrast to other measures of phase synchrony such as phase-locking value, which can only measure synchrony between two time-series, GFS is a global measure of neural phase synchrony that does not rely on the a priori selection of brain regions to be studied. When applied to EEG data, GFS has the added benefit of being reference-independent and more easily interpretable without the use of source models (Michels et al., 2012). Further research using the measure has found changes in GFS in those with Alzheimer’s disease and mild cognitive impairment (König et al., 2005), during REM sleep (Achermann et al., 2016), and during general anesthesia (Nicolaou & Georgiou, 2014).

To calculate GFS values for EEG, the data were first pre-processed. Following pre-processing, sensor-level EEG time-series were divided into non-overlapping, consecutive 2-second epochs. Each epoch was frequency transformed using a fast Fourier transform (FFT, Tukey window, taper sizeα=0.2), which yields the real and complex component of the signal of each electrode for each frequency value (1–40 Hz, step size = 0.1 Hz). These components are then plotted as vectors on the complex plane, with the magnitude of the vector representing the power of the signal at that frequency, the angle of the vector as measured from the real axis representing the phase, and the vector origin representing the reference of all EEG signals. Subsequently, a scatter plot of vector endpoints in the complex plane is generated for each frequency value, a representative of which can be seen inFigure 1B. The more these endpoints approximate a straight line, the more the signals are in phase or anti-phase. The more scattered the endpoints, the less the signals are in phase or anti-phase. To quantify the shape of the cloud formed by the vector endpoints, the points are entered into a two-dimensional principal component analysis, as principal components are orthogonal by definition. The resulting GFS value per epoch for a particular frequencyfis then determined by calculating the ratio of the eigenvalues ($\lambda_{1}(f)$and$\lambda_{2}(f)$) of these two principal components, as expressed inEquation 1.

$$GFS(f)=\frac{\left|\lambda_{1}(f)-\lambda_{2}(f)\right|}{\lambda_{1}(f)+\lambda_{2}(f)}$$ (1)

Finally, the overall GFS value for a particular frequency is obtained by taking the mean of the GFS values of all consecutive epochs for that frequency, creating a spectrum as shown inFigure 1B. To examine differences in GFS across age (using the Leipzig LEMON rsEEG dataset) and condition (ketotic vs. glycolytic), overall GFS values were categorized into their corresponding, classically-accepted frequency bands (da Silva, 2013), with endpoints adjusted to accommodate the filtering performed in pre-processing:δ(1–3.5 Hz),θ(4–7.5 Hz),α(8–13 Hz),β(14–30 Hz), lowγ(30–40 Hz), and broad (1–40 Hz).

### 2.4 Modeling effects of microscale parameter changes on global field synchronization

We used a conductance-based neural mass model (Breakspear et al., 2003) to test the effect of ion gradient dynamics and excitatory-inhibitory subpopulation coupling on GFS. This neural mass model has been previously validated to capture properties of fMRI and EEG resting-state activity (Endo et al., 2020) while incorporating both local ion dynamics and interregional connectivity. Following the mathematics of prior work (Endo et al., 2020), we built a 78-region whole-brain simulation using Neuroblox, a Julia library optimized for high-performance computing of dynamical brain circuit models (https://github.com/Neuroblox/Neuroblox.jl). Within the model, we varied ion (Na^+^, K^+^, Ca^2+^) gradients and conductances, intraregional connectivity (excitatory-excitatory/inhibitory), and interregional connectivity across a range of biophysically plausible values (Chesebro et al., 2023;Endo et al., 2020;Roberts et al., 2019). Ion parameters were changed because metabolic changes have been shown to alter ion dynamics in microscale experiments (Baeza-Lehnert et al., 2019). Excitatory-excitatory/inhibitory and global connectivity parameters were varied as a control to ensure that the effects in the model do not arise from just any changes to inter- or intra-regional connectivity. The ranges of the values changed can be found inTable 1. The whole-brain simulation for each parameter set was repeated with 10 different sets of initial conditions to sample across the distribution of simulation outcomes. The simulated membrane potentials of excitatory neurons were averaged within each region to generate 78 neural mass signals, which were transformed into EEG signals through multiplication with an average lead field (63 EEG channels×78 model ROIs) generated byEndo et al. (2020). Each 10-minute simulation was sampled at a rate of 1000 Hz and took∼2 minutes to generate. The first 1 minute of each simulated EEG signal was discarded before calculating GFS to allow the simulation to equilibrate.

**Table 1.** Parameter values used in the conductance-based neural mass model.

Parameter	Description	Default value	Variation range	Step size
**Nernst potentials**				
* $V_{\text{Na}}$	${\text{Na}}^{+}$ reversal potential	0.53	[0.42, 0.54]	0.005
* $V_{\text{K}}$	${\text{K}}^{+}$ reversal potential	-0.7	[-0.75, -0.6575]	0.0025
* $V_{\text{Ca}}$	${\text{Ca}}^{2+}$ reversal potential	1.0	[0.95, 1.01]	0.0025
$V_{\text{L}}$	Leak channels reversal potential	-0.5		
**Channel conductances**				
* $g_{\text{Na}}$	${\text{Na}}^{+}$ conductance	6.70	[6.6, 6.8]	0.01
* $g_{\text{K}}$	${\text{K}}^{+}$ conductance	2.00	[1.95, 2.05]	0.0025
* $g_{\text{Ca}}$	${\text{Ca}}^{2+}$ conductance	1.00	[0.95, 1.01]	0.0025
$g_{\text{L}}$	Leak channels conductance	-0.50		
**Channel voltage thresholds**				
$T_{\text{Na}}$	${\text{Na}}^{+}$ channel threshold	0.30		
$T_{\text{K}}$	${\text{K}}^{+}$ channel threshold	0.00		
$T_{\text{Ca}}$	${\text{Ca}}^{2+}$ channel threshold	-0.01		
**Channel voltage threshold variances**				
$\delta_{\text{Na}}$	${\text{Na}}^{+}$ channel threshold variance	0.15		
$\delta_{\text{K}}$	${\text{K}}^{+}$ channel threshold variance	0.30		
$\delta_{\text{Ca}}$	${\text{Ca}}^{2+}$ channel threshold variance	0.15		
**Excitatory & inhibitory population parameters**				
$V_{T}$	Excitatory neuron threshold voltage	0.00		
$Z_{T}$	Inhibitory neuron threshold voltage	0.00		
$\delta_{V,Z}$	Variance of excitatory/inhibitory thresholds	0.66		
$Q_{V_{\text{max}}}$	Excitatory population maximum firing rate	1.00		
$Q_{Z_{\text{max}}}$	Inhibitory population maximum firing rate	1.00		
* $a_{ee}$	Excitatory → excitatory synaptic strength	0.36	[0.33, 0.39]	0.005
* $a_{ei}$	Excitatory → inhibitory synaptic strength	2.00	[1.95, 2.05]	0.005
$a_{ie}$	Inhibitory → excitatory synaptic strength	2.00		
$a_{ne}$	Non-specific → excitatory synaptic strength	1.00		
$a_{ni}$	Non-specific → inhibitory synaptic strength	0.40		
**Other parameters**				
$I_{0}$	Subcortical excitatory input	0.30		
b	Time scaling factor	0.10		
ϕ	Temperature scaling factor	0.70		
$\tau_{\text{K}}$	${\text{K}}^{+}$ relaxation time	1.00		
$r_{\text{NMDA}}$	NMDA/AMPA receptor ratio	0.25		
c	ROI-to-ROI coupling constant	0.35		

The “Default Value” column lists the default parameter values used in the conductance-based neural mass model, followingEndo et al. (2020)andChesebro et al. (2023). If a parameter was varied to examine the effects on synchrony, it is labeled with an asterisk (^*^) in the first column and the range of parameter values tested is listed in the “Variation Range” column. Likewise, the step size used to sample between lowest and highest possible varied parameter value is listed in the “Step Size” column.

### 2.5 Brain network instability

Brain network instability (described for use with fMRI data inMujica-Parodi et al. (2020)) is a measure used to describe the persistence of brain networks over time. It can be considered a measure of dynamic functional connectivity (Hutchison et al., 2013), or a quantification of the frequency of switching between metastable brain states over various temporal scales (Roberts et al., 2019). To calculate brain network instability values for EEG, the data were first pre-processed as described above. Following pre-processing, sensor-level EEG time-series were divided into non-overlapping, consecutive epochs, or windows, with a 10 second window chosen for the results shown inFigures 2Cand2D. Within each window$W_{i}$an all-to-all, signed correlation matrix between all time-series is calculated, meaning these resulting matrices have a size ofn×n, withnbeing the number of clean channels remaining after pre-processing. Distance (in units of window size) between window pairs is decided by a value ofτchosen for the instability calculation. Instability is then calculated for each possible value ofτby taking the Frobenius, or L2, norm of the difference in correlation matrices of window pairs, and then taking the average of all norms. In the form of an equation, instability is written as

**Fig. 2.**
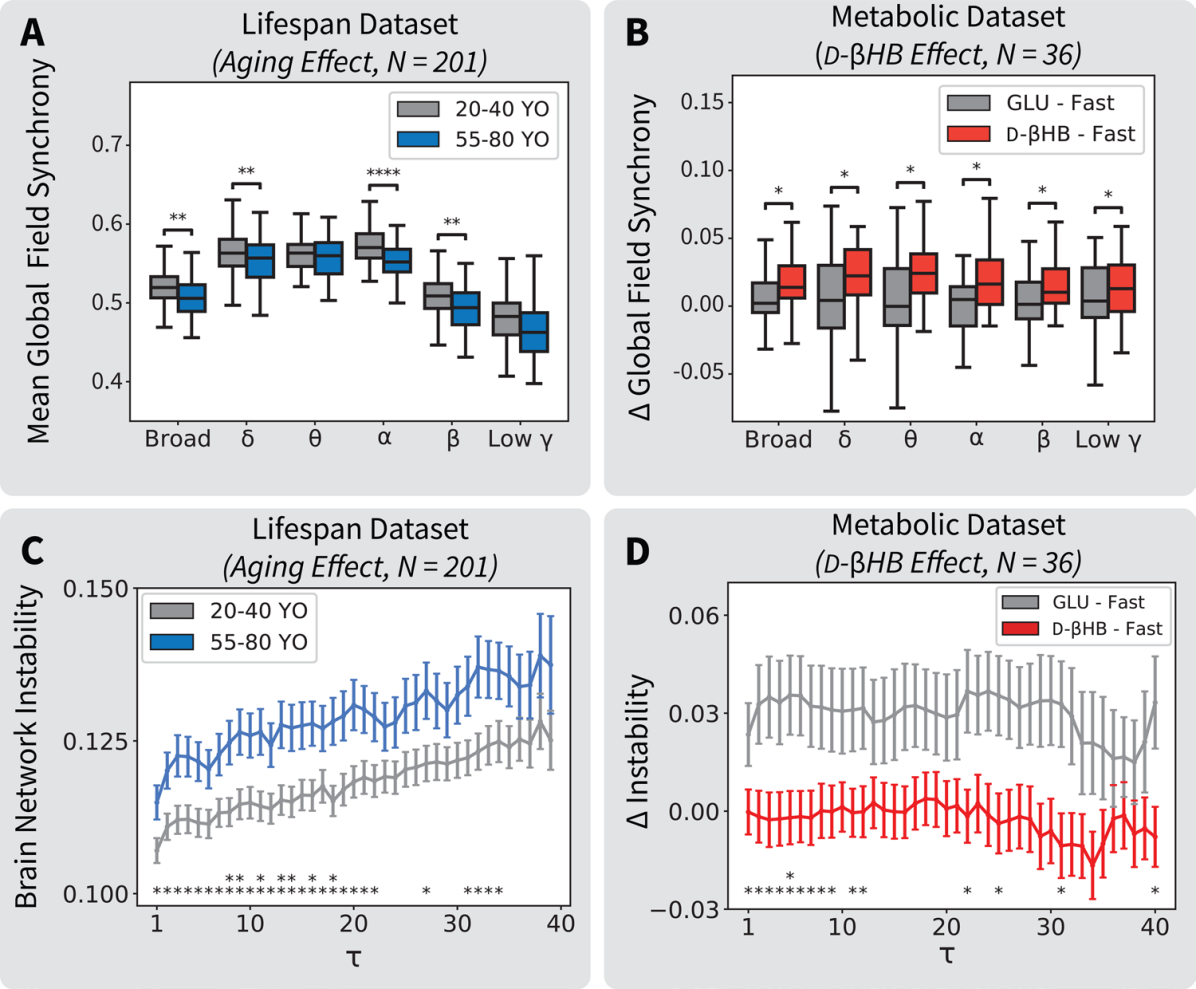
Global Field Synchronization, a global measure of neural synchrony, decreases with age and increases under ketosis. Brain network instability, a measure of dynamic functional connectivity, increases with age, and decreases under ketosis. (A) Mean synchrony is lower in the older (N = 63, 55 to 80 years) cohort than the younger (N = 138, 20 to 40 years) cohort in the broad,δ,α, andβbands of Leipzig’s LEMON EO, rsEEG scans. (B) Baseline-corrected synchrony is greater following acute administration ofD-βHB when compared to acute administration of glucose in all classically defined frequency bands of EO, rsEEG scans: broad,δ,θ,α,β, and lowγ, measured using a cohort of young, healthy individuals (N = 36,$\mu_{age}=26.9\pm 11.2$years). Descriptive statistics for (A) and (B) have been outlined inTable 2. (C) Brain network instability (window size = 10 seconds) is significantly greater in the older cohort than the younger cohort in the broad band of Leipzig’s LEMON EC, rsEEG scans for 27 out of 39 possible values ofτ, with p-values ranging from p=0.005to p=0.045and t-values ranging from t =2.023to t=2.846. When comparing across bandwidths, brain network instability (window size = 10 seconds,τ=1) of the older cohort is significantly greater in the broad (t=2.278, p=0.024),δ(t=3.581, p<0.001),θ(t=2.323, p=0.021), and lowγ(t=3.513, p<0.001) bands (all tested using independent t-tests). Error bars are SEM. When treating all values of instability perτper cohort as separate distributions, the mean instability in the older cohort is significantly greater than that of the younger cohort (mean difference = 0.011, p<0.001, t=10.277, tested using an independent t-test). (D) Baseline-corrected brain network instability (window size = 10 seconds) is significantly greater after acute administration of glucose when compared to acute administration of ketones in the broad (1–40 Hz) band of EC, resting-state EEG scans performed on a cohort of young, healthy individuals (N=36,$\mu_{age}=26.9\pm 11.2$years) for 15 out of 40 possible values ofτ, with p-values ranging from p=0.009to p=0.047and t-values ranging from t=2.055to t=2.744(all tested using paired t-tests). The stabilization of brain networks under ketosis was not dominated by a particular frequency band. Baseline correction for both metrics was performed by subtracting the pre-bolus (fasting) condition value from the post-bolus (either glucose orD-βHB) condition value within each respective metric. Error bars are SEM. When treating all values of instability perτper condition as separate distributions, the mean∆instability in the GLC–Fast condition is significantly greater than that of theD-βHB–Fast condition (mean difference = 0.032, p<0.001, t=33.031, tested using a paired t-test).

$$Instability\left(\tau ,\tau_{max}\right)=\frac{\sum_{i=1}^{\tau_{max}-\tau }\|corr\left(W_{i+\tau }\right)-corr\left(W_{i}\right){\|}_{F}}{\tau_{max}-\tau }$$ (2)

where$\tau_{max}$is the number of windows available, defined as the length of the time-series divided by the window size rounded down to the nearest multiple of the window size. For example, an EEG recording with a length of 55 seconds divided into window sizes of 10 seconds means$\tau_{max}\;=5$. Computing instability forτ=2for this example recording would yield

$$Instability\left(\tau =2,\tau_{max}=5\right)=\frac{\sum_{i=1}^{3}\|corr\left(W_{i+2}\right)-corr\left(W_{i}\right){\|}_{F}}{3}$$ (3)

$$=\frac{\|corr\left(W_{3}\right)-corr\left(W_{1}\right){\|}_{F}+\|corr\left(W_{4}\right)-corr\left(W_{2}\right){\|}_{F}+\|corr\left(W_{5}\right)-corr\left(W_{3}\right){\|}_{F}}{3}$$ (4)

When calculating instability, the window sizes within which correlation matrices are calculated and eventually subtracted from one another set a natural limit to the timescale at which changes in global network connectivity can be seen. As the temporal resolution of EEG is notably greater than that of fMRI, the window sizes used in the calculation of instability for the majority of this work are smaller than the windows used for our previous work (10 seconds as compared to 24 seconds) as more time points are available for calculation. By varying the window sizes used to calculate network instability, we determined that brain networks destabilize as a function of age only for networks whose detectable correlation differences persist for≥10 seconds (Fig. S2), and as such made the choice of window size to be 10 seconds for subsequent analyses. Furthermore, to ensure the increase in instability with age seen in the Leipzig LEMON dataset was not due to motion (as subject motion tends to increase with age (Seto et al., 2001)), we examined the relationship between the mean frame displacements (in mm) measured during the subjects’ corresponding rsfMRI scan and their brain network instability (τ=1, window size = 10 seconds) within the broad (1-40 Hz) frequency band of the subjects’ EC, rsEEG scan. We found no correlation between these two variables.

### 2.6 Statistics

Two-sided independent t-tests were used to determine whether mean GFS and instability differed significantly between the younger and older cohorts of the Leipzig LEMON rsEEG dataset within frequency bands (for GFS,Fig. 2A) and withinτvalues (for instability,Fig. 2C). Two-sided paired t-tests were used to determine whether mean GFS and instability differed significantly between the glucose and ketone conditions within frequency bands (for GFS,Fig. 2B) and withinτvalues (for instability,Fig. 2D). As instability values perτ(Fig. 2C, D) are by definition not independent, no multiple-comparisons correction was applied to these results. All other statistics (Fig. 2A, B) were corrected for multiple comparisons using the Benjamini–Hochberg procedure with a significance level set atα= 0.05.

### 2.7 Conductance-based neural mass model: single neural mass

Using the physiological and mathematical boundary conditions discussed inChesebro et al. (2023)to inform the model, the model equations are constructed as a system of three variables: mean excitatory membrane voltageV(t), mean inhibitory membrane voltageZ(t), and the proportion of potassium channels open at a given timeW(t). Note that whileV(t),Z(t), andW(t)are all time-dependent, we omit this dependence in the following equations for ease of reading. Given this understanding, the neural mass model is defined as:

dVdt=−{gCa+rNMDAaeeQV}mCa(V−VCa)−{gNamNa+aeeQV}(V−VNa)−gKW(V−VK)−gL(V−VL)−aieZQZ+aneI0 (5)

$$\frac{dZ}{dt}=b\left(a_{ni}I_{0}+a_{ei}VQ_{V}\right)$$ (6)

$$\frac{dW}{dt}=\phi \frac{m_{\text{K}}-W}{\tau_{\text{K}}}$$ (7)

In these equations,$Q_{V}$and$Q_{Z}$are the mean firing rates for excitatory and inhibitory cell populations, respectively. These are computed as

$$Q_{V}=0.5Q_{V_{\text{max}}}\left(1+\text{tanh}\frac{V-V_{T}}{\delta_{V}}\right)$$ (8)

$$Q_{Z}=0.5Q_{Z_{\text{max}}}\left(1+\text{tanh}\frac{V-V_{Z}}{\delta_{Z}}\right)$$ (9)

The individual ion channel gating functions ($m_{\text{Na}}$,$m_{\text{K}}$and$m_{\text{Ca}}$) take the form

$$m_{\text{ion}}=0.5\left(1+\text{tanh}\frac{V-T_{\text{ion}}}{\delta_{\text{ion}}}\right)$$ (10)

where$m_{\text{ion}}$is the fraction of voltage-dependent channels open at any given time. Default values and descriptions for all constants in these equations are given inTable 1. Note that parameter values are unit-less to scale to a reasonable modeling range (i.e.,V,Q∈(−1,1)andW∈(0,1)), and the integration time stepdtis in milliseconds.

We note that the three ions of interest are modeled in three different manners. Sodium serves as the dominant shape determinant of the neural mass spiking activity as it has the highest net positive conductance coupled to its ion channel gating function. Calcium serves as a secondary support of the spiking activity, providing some of the amplitude to the spiking activity. However, because the calcium gating function is also coupled to the excitatory population firing rate and the ratio of NMDA/AMPA receptors, calcium more importantly provides feedback to the neural mass firing rate. Finally, due to its more detailed modeling as a separate differential equation, potassium plays a unique role in determining the frequency of spiking activity (at larger reversal potentials) and the duration of the refractory period (at smaller reversal potentials). As a consequence of this extra modeling step, potassium also has a different ion gradient landscape than sodium and calcium.

While the excitatory (pyramidal) cell populationV(t)is modeled using the ion dynamics described above, the inhibitory populationZ(t)is a purely phenomenological model, receiving only the excitatory input via$a_{ei}$and a background current via$a_{ni}$. Although this serves to model the relationship between inhibitory interneurons and the excitatory pyramidal cells (as inLarter et al. (1999)), it does imply a caveat when interpreting the effects of altered ion gradients.

Since the model is a hybrid of a biophysically detailed excitatory neuron population and a phenomenological inhibitory population, claims regarding how closely this model resembles true biological neurons are necessarily constrained. However, the advantage of this neural mass model is that it produces physiologically interesting dynamics (e.g., burst-spiking) that are more common in next-generation neural mass models (Taher et al., 2022) than in traditional (e.g., Wilson-Cowan) oscillatory models.

### 2.8 Conductance-based neural mass model: coupled neural masses

Equations 5–7describe a single neural mass comprising two subnetworks. Coupling between pairs of neural masses (iandj) can also be achieved through connection terms:

$$cQ_{i}^{\text{network}}=c\frac{\sum_{j}u_{i,j}Q_{V_{j}}}{\sum u_{i,j}}$$ (11)

Here,cis the coupling constant,$Q_{V_{j}}$is the mean excitatory firing rate of regionj, and$u_{i,j}$is the strength of connection between regionsiandj. To ensure that overall input current is approximately constant, the balancing between interregional and self-coupling takes the form of competitive agonism, wherecis the weight of interregional coupling and(1−c)is self-coupling. The associated multi-regional neural mass model equations are then given by:

dVidt=− {gCa+rNMDAaee[(1−c)QV+ cQinetwork]}mCa(Vi−VCa)−{gNamNa+ aee[(1−c)QV+cQinetwork]}(Vi−VNa)− gKWi(Vi−VK)−gL(Vi−VL)− aieZiQZ+aneI0 (12)

$$\frac{dZ_{i}}{dt}=b\left(a_{ni}I_{0}+a_{ei}V_{i}Q_{V}\right)$$ (13)

$$\frac{dW_{i}}{dt}=\phi \frac{m_{\text{K}}-W_{i}}{\tau_{\text{K}}}$$ (14)

In this work, we useEquations 12–14, varying the microscale parameters (Table 1) therein to observe the effects on macroscale neural synchrony. Following prior work (Endo et al., 2020), we computed a 78 region whole-brain model. To determine connectivity between regions, we used a DTI-derived structural connectivity map averaged across healthy individuals. This connectivity has previously been shown to produce simulations with the conductance-based neural mass model used in this work that are consistent with both EEG and fMRI scale measurements (Endo et al., 2020). This map is the same as inEndo et al. (2020), having been provided by the authors as supplemental data. This is the standard approach employed by toolboxes such as The Virtual Brain (Sanz Leon et al., 2013).

## 3 Results

### 3.1 Age modulates neural signaling

Using the Leipzig LEMON rsEEG dataset, a comparison of Global Field Synchronization (GFS, see Methods) between the younger (aged 20 to 40 years, N = 138) and older (aged 55 to 80 years, N = 63) cohorts within classically defined frequency bands (δ(1–3.5 Hz),θ(4–7.5 Hz),α(8–13 Hz),β(14–30 Hz), lowγ(30–40 Hz), and broad (1–40 Hz), (da Silva, 2013)) showed age to decrease neural synchrony in the broad,θ,α, and lowγbands (Fig. 2A,Table 2). Likewise, older brains showed destabilization of their networks (Fig. 2C).

**Table 2.** Descriptive statistics of data underlyingFigures 2Aand2B.

Lifespan dataset (N = 201)			
Band	t	p (corr.)	Mean Difference
Broad	-3.260	0.004	-0.0126
δ	-3.001	0.005	-0.0130
θ	-1.097	0.274	-0.004
α	-5.119	4.33e-06	-0.018
β	-2.965	0.005	-0.014
Low γ	-1.923	0.067	-0.011

Metabolic dataset (N = 36)			
Band	t	p (corr.)	Mean difference
Broad	2.659	0.018	0.029
δ	2.763	0.018	0.035
θ	3.184	0.018	0.036
α	2.693	0.018	0.028
β	2.331	0.031	0.027
Low γ	2.235	0.032	0.027

For the*Lifespan Dataset*, independent t-tests (with FDR correction) were performed to test for mean differences between the older (55–80 YO) and younger (20–40 YO) cohorts. For the*Metabolic Dataset*, paired t-tests (with FDR correction) were performed to test for mean differences inΔGFS between theD-βHB–Fast and GLC–Fast conditions.

### 3.2 Metabolism modulates neural signaling

Changes in GFS values (ΔGFS) for the metabolic study cohort (N = 36,$\mu_{age}\;=$26.9±11.2 years) were calculated by subtracting the average fasting condition GFS from the average experimental (glycolytic or ketotic) condition GFS within each band. Comparing the pre- and post-bolus synchrony values showed decreased synchrony after administration of the glucose bolus and increased synchrony after administration of the ketone bolus for all frequency bands in the rsEEG eyes-open (EO) condition (Fig. 2B,Table 2). Following glucose challenge, brain networks destabilized; in contrast, following ketone challenge, brain networks stabilized (Fig. 2D) in rsEEG. Both results were observed in the broad (1–40 Hz) frequency band of eyes-closed (EC) rsEEG.

### 3.3 Identifying candidate mechanisms using multiscale modeling

**Fig. 3.**
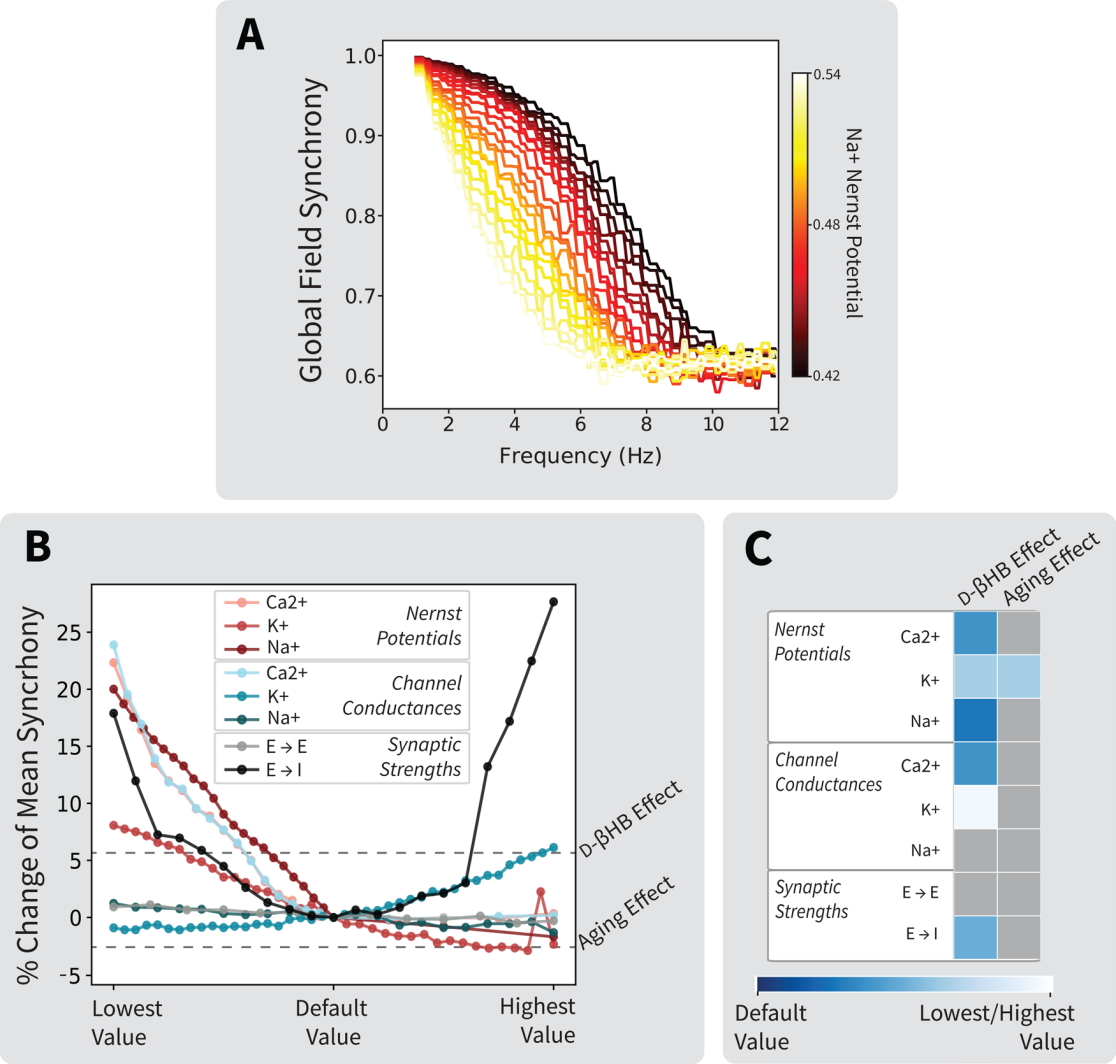
Variation of the K^+^Nernst potential within a conductance-based neural mass model uniquely predicts changes in Global Field Sychrony (GFS) seen in aging and following acute administration ofD-βHB. (A) An example of the GFS spectra for simulated EEG data generated using a conductance-based neural mass model. The color indicates the value of the Na^+^Nernst potential used in the simulation. (B) Individually varying parameters within the model above and below their default values leads to changes in GFS. Parameters varied are color-coded by class (Nernst potentials, channel conductances, and excitatory→excitatory/inhibitory synaptic strengths). (C) Heatmap of the relative parameter change needed to replicate the magnitude of changes seen in broadband GFS (Fig. 2A, B).

We next sought to identify the mechanistic basis for age and metabolism-related changes observed in rsEEG GFS. To do so, we used a bottom-up approach by varying microscale parameters in the neural mass model to generate 2082 simulated rsEEG signals, from which we computed GFS and instability. We observed high sensitivity of GFS to changes in all (Ca^2+^, K^+^, and Na^+^) Nernst potentials, Ca^2+^and K^+^channel conductances, and excitatory→inhibitory synaptic strength (Fig. 3B). Only variation of the K^+^Nernst potential within this model explained both the magnitude of the changes seen with older age (2.5% decrease) and followingD-βHB consumption (5.6% increase) in broadband GFS (Fig. 3C). Sensitivity of brain network instability to the parameter changes was binary: either it was not sensitive to changes in parameters, or network instability changed in non-consistent ways and with large magnitudes that did not reflect the smaller magnitudes seen in human data (Fig. S1). Furthermore, brain network instability was found to be uncorrelated with GFS within the broad (1-40 Hz) frequency band of Leipzig’s LEMON rsEEG dataset (p = 0.090,$r^{2}$= 0.014, N = 201).

## 4 Discussion

It has been hypothesized that brain aging results from an “energy crisis” in the brain, in which decreased glucose oxidation capability leads to constrained ATP availability for neurons (Jensen et al., 2020;Hoyer, 1982), disrupting neuronal signaling and thus dysregulating the neural circuits that underlie cognitive processing. The fact that metabolism modulated both our EEG- and fMRI-derived biomarkers for brain aging provides robust support for this hypothesis. Our findings, based on a conductance-based neural mass model, suggest that the dysregulation of K^+^ion gradients, crucially governed by ATP, is the primary driver of the observed changes in brain-wide EEG synchronization.

Dysfunction of Na^+^/K^+^-ATPase causes depolarization of the membrane potential, and thus desynchronization between brain regions (Chesebro et al., 2023). Ion pumps are a sink of metabolic resources in the brain, and thus Na^+^/K^+^-ATP-ase dysregulation is also expected under metabolic constraints, which would further exacerbate ion gradient dysregulation (Baeza-Lehnert et al., 2019). Changes in potassium reversal potential have been implicated in a number of different age-related processes (Scott et al., 1988;Sesti et al., 2010). The depletion of the potassium gradient can stem from damage to potassium channels by reactive oxygen species (Sesti et al., 2010) or from calcium signaling dysregulation (Farajnia et al., 2015;Power et al., 2002), or by a combination of accumulated insults, leading to impairment.

This conductance-based neural mass model provides a biophysically detailed simulation of ion dynamics while allowing for a whole-brain simulation by abstracting away population details. While this derivation preserves local ion dynamics (Larter et al., 1999), it does not fully capture emergent properties of neural populations (e.g., the model preserves cortical wave dynamics (Roberts et al., 2019), but not power spectra). We found that GFS was sensitive to changes in the biophysical model parameters (Fig. 3B), reflecting changes that were on the same scale of magnitude as the changes in GFS seen in the human (non-simulated) data. However, the absolute magnitude of the spectra produced using our modeling approach did not match that of spectra produced from our human rsEEG data. Furthermore, brain network instability was either not sensitive at all to changes in parameters, or for those parameters that did alter network instability, changed it in non-consistent ways and with large magnitudes of change that did not accurately reflect the smaller magnitudes seen in human data (Fig. S1). These limitations may be due to the inability of this neural mass model to capture certain emergent properties of neural populations and have been partially addressed in next-generation neural mass models (Byrne et al., 2020). Brain network instability and neural synchrony were found to be uncorrelated, implying the two metrics quantify distinct neural features, of which the features of the latter are better captured by the conductance-based neural mass model used in this work. There are several potential advances in the field which may lead to better capturing of both neural features. Some examples are the incorporation of networks of firing neurons that model ion and metabolic kinetics to existing models (Dutta et al., 2023), including additional metabolic variables when considering the BOLD signal fluctuations (as is becoming common in systems pharmacology models (Spiros et al., 2014)), and the development of next-generation neural mass models that follow a more “human-like” power spectrum (Byrne et al., 2020). As of yet, however, these models do not fully incorporate the same biophysical detail coupled with scalability as this conductance-based neural mass model does. Fusing these approaches is a topic of current interest in the field of computational neuroscience.

Prior literature has established ion gradient regulation to be the most significant energy sink within the brain (Baeza-Lehnert et al., 2019;Meyer et al., 2022). This is consistent with our results linking emergent whole-brain network effects to ion gradient regulation. Our further isolation of the consistent candidate mechanisms to K^+^Nernst potentials is also in agreement with*in vitro*experimental evidence that ketosis directly impacts neuronal K^+^regulation (Ma et al., 2007). In conclusion, our findings indicate promising avenues for future research, directly examining the association between age-related hypometabolism and disrupted neuronal K^+^gradients, and their reversal by ketosis. Furthermore, the experimental validation of our findings highlights the role of generative models, guided by clinical neuroimaging, in connecting molecularly targeted therapies to patient outcomes while simultaneously showcasing their utility in mitigating confirmation bias by considering multiple hypotheses simultaneously.

## Supplementary Material

Supplementary Material

## Data Availability

Data and code are available at our website:https://www.lcneuro.org/data/eeg. Leipzig LEMON data are publicly available athttp://fcon_1000.projects.nitrc.org/indi/retro/MPI_LEMON.html.
