# Supplementary material for: Ketosis regulates K+ ion channels, strengthening brain-wide signaling disrupted by age
Source: Imaging Neurosci (Camb). 2024 May 8;2:imag-2-00163. doi: 10.1162/imag_a_00163 (PMC11633768; doi:10.1162/imag_a_00163)
Supplement: Supplementary Material [file imag_a_00163-supp.pdf]

Supplementary Information

Clinical and Demographic Information of Metabolic Study Participants

**Measure**		
**Sex**		N = 36 (18 female)
**Age (years)**	Average	26.9 $\pm$ 11.2
	Median	21.5
	Range	19 - 65
**Ethnicity**	White	16
	Black	1
	Asian	15
	Hispanic	4
**HbA1c (%)**	Average	5.1 $\pm$ 0.4
	Median	5.1
	Range	4.4 - 6.1

Table S1. Clinical and Demographic Information of the Metabolic Study Participants.

Justification of Choice of Neural Synchrony as Macroscale Biomarker for Top-Level of Multi-Scale Model

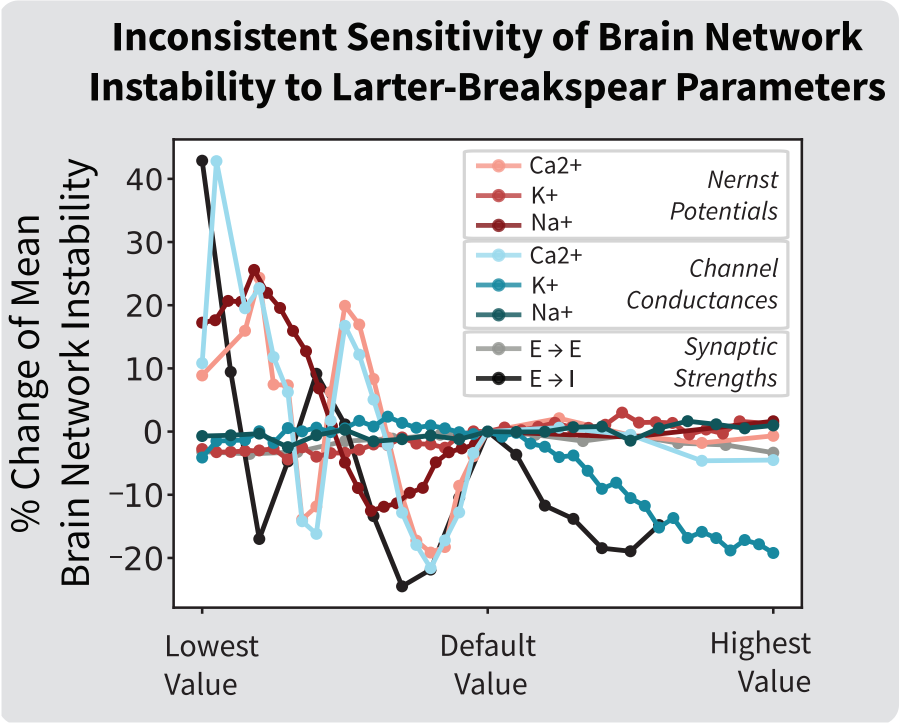

Figure S1. Modeling brain network instability using simulated rsEEG produced by the Larter-Breakspear neural mass model shows inconsistent sensitivity of network instability to model parameter variations.

Effect of Window Size on Brain Network Instability Calculation

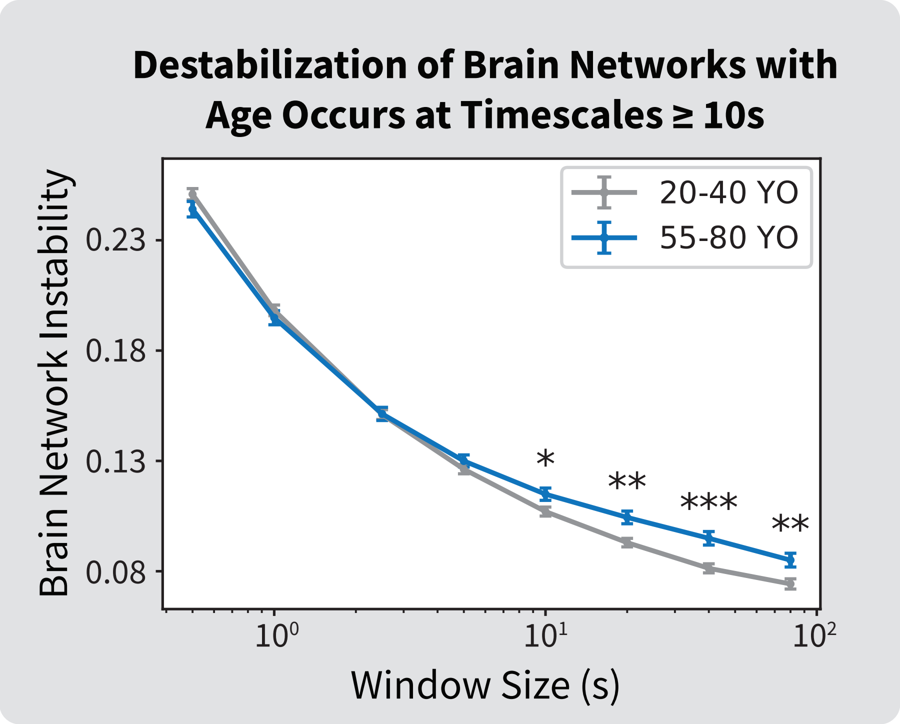

Figure S2. High temporal resolution of EEG reveals timescale of age-related brain network destabilization. (A) One advantage of EEG over fMRI is a more than 3,000-fold increase in temporal resolution of the measured signal. Taking advantage of this high temporal resolution, we calculated brain network instability using Leipzig LEMON's younger (N = aged 20 to 40 years) and older (aged 55 to 80 years) eyes-closed, rsEEG cohorts for a variety of window sizes ranging from 0.5 s to 80 s. We found that network instability is significantly greater in the older cohort for window sizes $\geq$ 10 s.
